# Echocardiography and Electrocardiography Variables Correlate With the New York Heart Association classification: An Observational Study of Ischemic Cardiomyopathy Patients

**DOI:** 10.1097/MD.0000000000007071

**Published:** 2017-06-30

**Authors:** Ying Hu, Shifeng Jiang, Siyuan Lu, Rong Xu, Yunping Huang, Zongliang Zhao, Yi Qu

**Affiliations:** a Department of Geriatrics, Xuhui District Central Hospital; b Department of Geriatrics, Qingpu Branch of Zhongshan Hospital, Fudan University; c Geriatric Nursing Services, Xuhui District Tianlin Street Community Health Service Center General, Shanghai, China.

**Keywords:** cardiac function, cardiac output, echocardiography, electrocardiography, ischemic cardiomyopathy, NYHA

## Abstract

The aim of our study was to determine whether combinations of ultrasound echocardiography (UCG) and electrocardiography (EKG) parameters correlated with the functional status of ischemic cardiomyopathy (ICM) patients according to the New York Heart Association (NYHA) classification system.

We assessed 536 elderly Chinese ICM patients according to the NYHA criteria, which included 196 patients with type 2 diabetes mellitus (T2DM). All of the patients underwent UCG. Transmural dispersion of ventricular repolarization was examined using EKG. Cumulative odds logistic regression was performed to evaluate associations between NYHA class and the demographic, clinical, UCG, and EKG variables based on the odds ratio (OR) and 95% confidence interval (CI). A Pearson analysis was also performed to examine correlations between the NYHA classification and the UCG and EKG variables.

Based on the NYHA assessment, 140, 147, 138, and 111 patients were identified as class I, II, III and IV, respectively. A comparison of UCG and EKG variables based on T2DM status showed that CO and Tp-e differed significantly between all NYHA classes (*P* < .05 for all), with values of each increasing with increasing NYHA class regardless of T2DM status. Multivariate logistic regression analysis showed that the disease course (OR: 1.30; 95% CI: 1.20–1.40), heart rate (OR: 1.16; 95% CI: 1.12–1.21), T wave peak to endpoint (Tp-e; OR: 1.22; 95% CI: 1.18–1.27), dispersion of the QT interval (OR: 0.98; 95% CI: 0.95–1.22), left ventricular fractional shortening (OR: 0.82; 95% CI: 0.78–0.87), cardiac output (CO; OR: 5.58; 95% CI: 3.08–10.13) were significantly associated with the NYHA class (*P* < .0001 for all). A Pearson correlation analysis showed that Tp-e (*r* = 0.75982, *P* < .0001), CO (*r* = 0.56072, *P* < .0001), and stroke volume (*r* = −0.14839, *P* = .0006) significantly correlated with the NYHA class.

An index consisting of Tp-e and CO will be useful for corroborating the results of the NYHA assessment of ICM patients.

## Abstract 2

Supplemental Digital Content is available in the text

## 1 Introduction

Cardiovascular disease is a major contributor to the global disease burden.^[1,2]^ Among these, ischemic cardiomyopathy (ICM) is highly prevalent. The estimated worldwide prevalence of ICM is 26 million ^[2]^, with the global incidence of ICM being 195.3 per 100,000 in men and 115.0 per 100,000 in women.^[1]^

An important aspect when planning the treatment of ICM is determining the extent to which cardiac function has been compromised. The New York Heart Association (NYHA) classification system is widely used to indirectly assess the effects of ICM on cardiac function by categorizing patients based on their limitations in carrying out routine physical activity.^[3–5]^ Although the validity of the NYHA classification has been confirmed^[6]^, it has demonstrated suboptimal reproducibility and a lack of sensitivity for detecting clinically important variations, which are thought to stem from the subjective nature of the NYHA criteria and self-reported patient symptoms.^[7,8]^ The functional status of ICM patients, as determined by the NYHA classification, is distinct from their cardiovascular performance and exercise capacity, which are assessed on more objective clinical criteria.^[9,10]^

Ultrasound-based echocardiography is widely used for clinical assessment and provides data regarding cardiac anatomy and ventricular function that are altered in various cardiac disorders, including cardiomyopathy.^[11–14]^ Dynamic changes in features of surface electrocardiography (EKG) can also reveal various pathological conditions affecting the electrophysiology of the heart.^[15–17]^ A prolonged interval between the peak and the end of the T wave represents transmural dispersion of ventricular repolarization (TDR).^[18]^ Factors that delay conduction between epicardial cells and M cells during epicardial stimulation, such as damage to cardiomyocytes resulting from an ischemic event, are thought to amplify TDR.^[19,20]^ Greater heterogeneity in TDR has been reported in patients with type 2 diabetes mellitus (T2DM) following myocardial infarction (MI), compared with post-MI patients without T2DM.^[21]^

Given that ischemic cardiac injury is associated with perturbations in UCG and EKG parameters, we reasoned that such changes might correlate with the level of functional impairment experienced by patients following MI. The aim of the present study was to determine whether combinations of UCG and EKG measurements correlated with the NYHA-based functional status of ICM patients. We also compared the UCG and EKG features with NYHA assessment in patients with T2DM to determine whether the effects of T2DM and cardiomyopathy influenced the relationship between UCG and EKG features and the cardiac functional status according to NYHA.

Though the NYHA classification is an easy and convenient method, it has certain limitations in that it is difficult to confirm the patient's subjective information. Furthermore, sometimes misdiagnosis may occur in patients with similar symptoms of heart failure, but with normal cardiac function, or when the patients look healthy but cardiac function has declined. In addition, the NYHA classification lacks quantitative estimations and may lead to a physician's judgment based on their subjective knowledge. The evaluation is also susceptible to the influence of non-heart failure symptoms diagnosed as heart failure indications. Therefore, this study was performed to find quantifiable variables that may correlate with the NYHA classification. Our results showed that certain UCG and EKG parameters correlated with the NYHA classification of ICM patients, suggesting that these factors might be useful objective measures for corroborating the NYHA classification.

## 2 Materials and Methods

### 2.1 Study population

The study was approved by the Research Ethics Committee of Xuhui Medical Center, and was performed in accordance with the guidelines of the Helsinki Declaration with regard to ethical principles for research involving human subjects. Written informed consent was obtained from all of the participants before enrollment in the study. Patients receiving outpatient or inpatient treatment for ICM at our institution between January 1 and December 31, 2015, were reviewed for enrollment. The inclusion criteria were: 65 years of age or older; normal sinus rhythm; previous diagnosis of acute MI according to the diagnostic criteria of the World Health Organization (WHO)^[22]^ or a previous diagnosis of coronary heart disease confirmed by angiography; and a previous diagnosis of chronic heart failure according to the national diagnostic criteria for China, which are a modified version of those suggested by the WHO.^[23]^ Patients meeting any of the following criteria were excluded: atrial fibrillation; electrolyte disorders; bundle branch block; used drugs that influence the QT interval within 2 weeks of enrollment, such as monoamine oxidase inhibitors, class-I or class-III anti-arrhythmic drugs, or non-double hydrogen dihydropyridine calcium antagonists; uncontrolled severe diabetes; hyperthyroidism; a diagnosis of pre-excitation syndrome, or serious liver or kidney dysfunction. An equal number of men and women who qualified for participation in the study were randomly selected based on the admission sequence number.

### 2.2 Clinical and demographic variables

One day after the enrollment date, 2 mL of blood was collected by venipuncture into EDTA-coated anticoagulant tubes after the patient had fasted overnight. A 250-μL blood sample from each patient was rapidly analyzed in a Biosite Triage diagnostic instrument (Alere, Waltham, MA). Age, sex, body mass index (BMI), course of disease, blood pressure, heart rate (HR), levels of serum lipids, the blood glucose level, currently used medication, and complications were recorded for each patient using a standardized questionnaire.

### 2.3 Cardiac functional status assessment

On the day following enrollment, each patient was categorized independently by 2 investigators (RX and YH) as class I, II, III, or IV according to NYHA guidelines as previously described.^[5]^ Any conflicts were resolved based on the judgment of a third investigator (SJ).

### 2.4 UCG assessment

Two-dimensional color Doppler ultrasound was performed by a qualified researcher, using an IE33 Ultrasound System (Philips Healthcare, Andover, MA), immediately after blood analysis. The left ventricular internal diameter at the end of diastole (LVIDd), stroke volume (SV), cardiac output (CO), left ventricular ejection fraction (LVEF), left ventricular fractional shortening (FS), and the ratio of the peak mitral blood flow velocity during early diastole to that during atrial contraction (E/A) were determined using the Simpson method.^[24]^

### 2.5 EKG assessment

Standard resting 12-lead body-surface EKG was recorded for each patient on the day following enrollment and >6 months later using a CardiMax FX-7302 EKG recorder (Fukuda Denshi, Tokyo, Japan), with a paper speed of 25 mm/s and the gain set at 10 mm/mV to ensure a clear, stable baseline with no interference. The interval from the peak to the end of the T wave was defined as Tp-e, and the Q to T interval (QT) as the interval between the first deflection of the QRS complex and the end of the T wave. QT was recorded by all 12 leads and corrected based on HR using the Bazett formula to obtain the corrected QT (QTc).^[25]^ The dispersion of the QT interval (QTd) was defined as the difference between the longest and shortest QT interval recorded by different leads. The HR, QTc, QTd, Tp-e, and Tp-e/QTc were determined in 3 continuous cardiac cycles by the same investigator from each of the 2 EKGs recorded for each patient.^[21]^

### 2.6 Statistical analysis

The statistical analysis was performed using SPSS for Windows (Version 13.0, SPSS Inc, Chicago, IL). A descriptive analysis of the demographic, clinical, EKG, and UCG variables was performed, for which continuous variables were reported as the mean ± standard error and categorical variables as the number of observations (n) and the percentage of the total for the cohort or subgroup. All the potential difference comparisons of continuous variables were carried out using analysis of variance, the difference between 2 groups using the Student-Newman-Keuls test, and intergroup differences of categorical variables evaluated using *χ*^2^ or Fisher exact tests. Univariate cumulative odds logistic regression was performed in which the NYHA classes I through IV were treated as the dependent variables and each of the demographic, clinical, EKG, and UCG variables were treated as independent variables. The probability of the NYHA outcome was calculated as the odds ratio (OR) and 95% confidence interval (CI). This evaluation was followed by a multivariate cumulative odds logistic regression analysis in which NYHA class I to IV was treated as the dependent variables. Correlations between the NYHA class and the UCG and EKG variables were evaluated using a Pearson correlation method. The level of statistical significance for all analyses was set at *P* < .05.

## 3 Results

### 3.1 Demographic and clinical characteristics of the NYHA classes

The demographic and clinical characteristics of the ICM patients are presented in Table 1. A total of 536 patients were included in the study. The distribution of the patients in the NYHA classes was as follows: class I (n = 140, 26.1%), class II (n = 147, 27.4%), class III (n = 138, 25.7%), and class IV (n = 111, 20.7%). No significant difference in sex or serum lipid levels was detected based on NYHA the class (*P* = .66 and *P* = .07, respectively). Age differed significantly between the classes (*P* < .000), with the median age increasing with increasing class level. The class-IV patients had a significantly longer course of disease (20.00 ± 8.00 years) and higher HR (95.00 ± 18.00 beats/min), compared with those of the other groups (*P* < .000 for both). Although BMI varied significantly between the NYHA classes (*P* < .000), no clear trend in BMI was observed. Patients with T2DM comprised 36.6% (n = 196) of the study cohort, and the number of T2DM patients varied significantly, based on the NYHA class (*P* < .000).

**Table 1:**
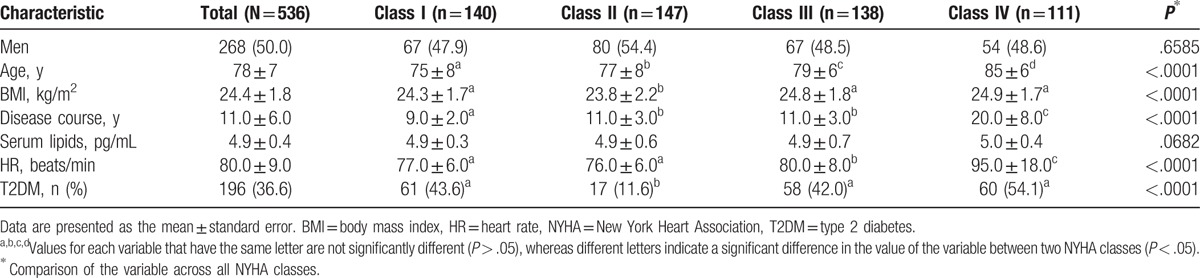
Comparison of demographic and clinical data of ischemic cardiomyopathy patients based on New York Heart Association class.

### 3.2 Comparison of UCG and EKG parameters between NYHA classes

In the comparison of EKG parameters based on the NYHA class, the values for Tp-e, QTc, QTd, and Tp-e/QTc varied significantly (*P* < .000; Table 2), with trends toward significantly higher values with higher classes observed for Tp-e and CO in particular. Only Tp-e and Tp-e/QTc differed significantly (*P* < .000) between increasing NYHA classes in non-T2DM patients, and only Tp-e differed significantly (*P* < 0.000) between increasing NYHA classes in T2DM patients (see Table S1 in Supplemental Digital Content showing subgroup analysis of EKG parameters based on T2DM status). In the analysis of UCG parameters, we found that FS and LVEF exhibited decreasing trends as the NYHA class decreased and that LVIDd and CO exhibited higher values as the NYHA class decreased (Table 2). Although all of the UCG parameters in both T2DM and non-T2DM patients differed significantly across all NYHA classes, only CO differed significantly for each of the NYHA classes, with a higher CO observed with increasing NYHA class in both T2DM and non-T2DM patients. These data suggest that CO might be a useful indicator of NYHA class in both T2DM and non-T2DM patients (see Table S2 in Supplemental Digital Content showing subgroup analysis of UCG parameters based on T2DM status).

**Table 2:**
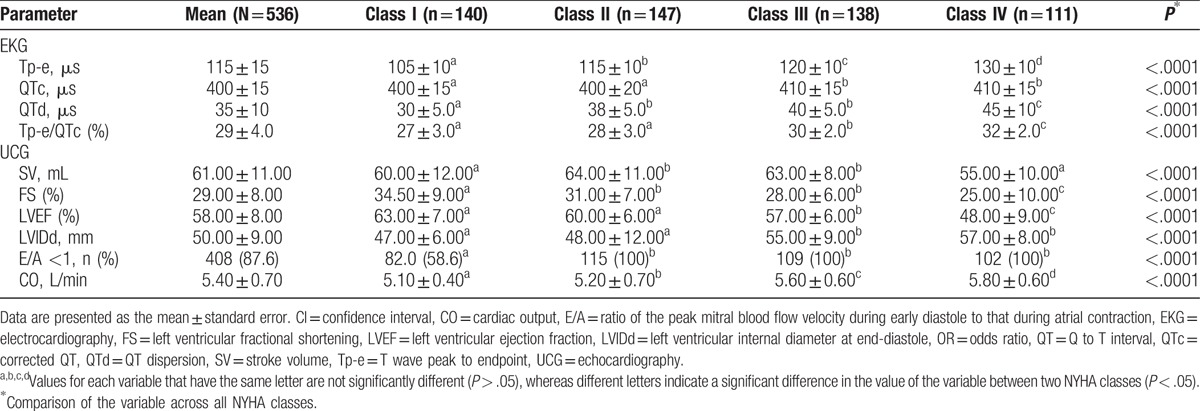
Comparison of electrocardiography and echocardiography variables based on New York Heart Association class.

### 3.3 Multiple UCG and EKG variables are associated with NYHA class

The univariate cumulative odds logistic regression analysis showed that age, BMI, disease course, T2DM status, HR, Tp-e, QTc, QTd, Tp-e/ QTc, LVIDd, and CO significantly correlated with the NYHA classes (*P* < .05 for all; Table 3), whereas the serum lipid level and sex (based on OR = 1 for men) were not significantly associated with the NYHA class. The multivariate cumulative odds logistic regression analysis showed that the disease course, HR, Tp-e, QTd, FS, and CO were significantly associated with the NYHA class (Table 4).

**Table 3:**
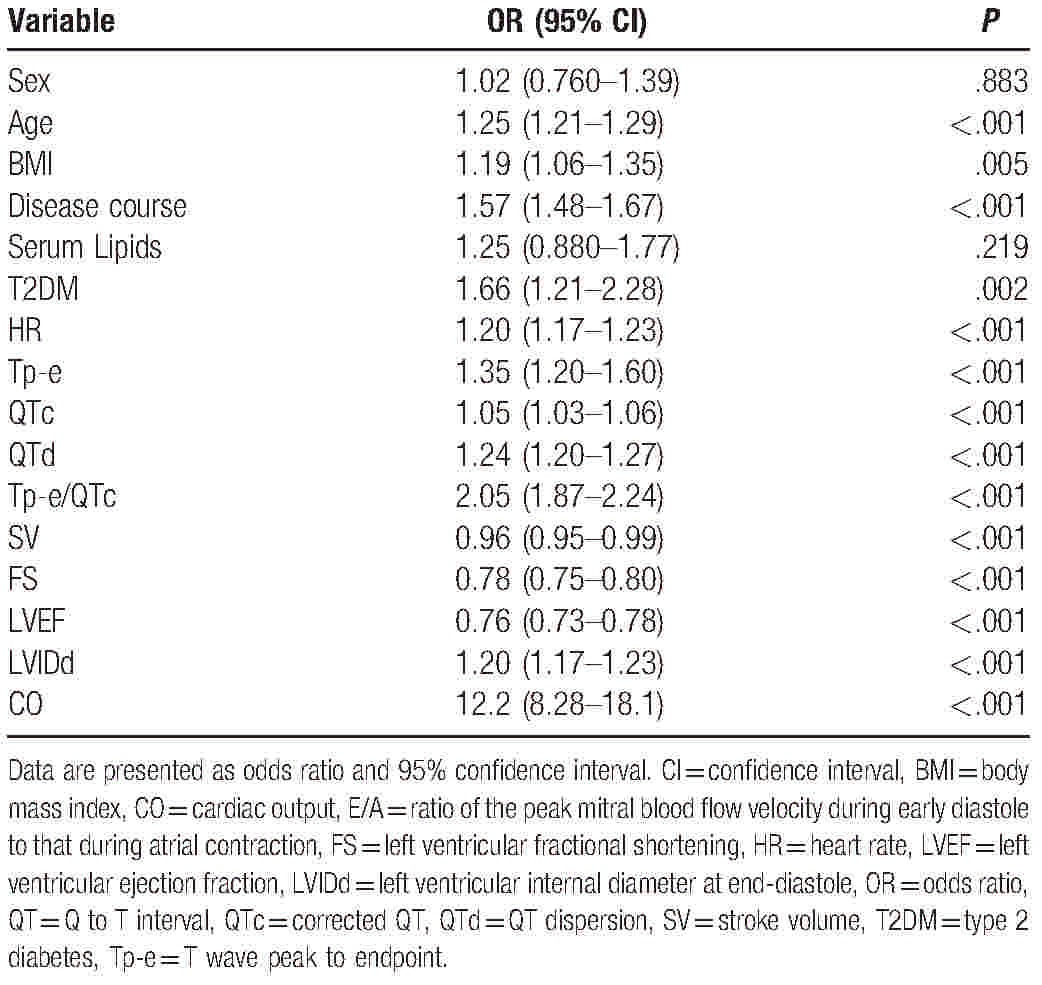
Univariate cumulative odds logistic regression analysis of associations between New York Heart Association class and the demographic, clinical, electrocardiography, and echocardiography variables of ischemic cardiomyopathy patients.

**Table 4:**
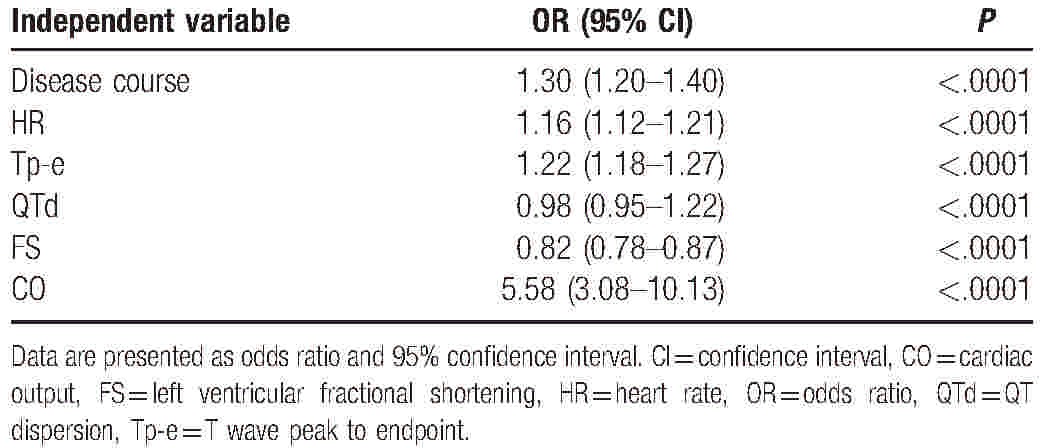
Multivariate cumulative odds logistic regression analysis of associations between New York Heart Association class and the clinical, electrocardiography, and echocardiography variables of ischemic cardiomyopathy patients.

### 3.4 UCG and EKG variables correlate with NYHA class

Correlations between NYHA class and the UCG and EKG variables were evaluated using a Pearson correlation method. Data plots for the Pearson analysis are shown in Figure S1 in the Supplemental Digital Content. Based on the trends observed in the data presented in Table 2, Tp-e, CO, and SV were selected for the Pearson correlation analysis. As shown in Table 5, we found that Tp-e, CO, and SV significantly correlated with the NYHA class (*r* = 0.75982, *r* = 0.5607, and *r* = −0.14839, respectively; *P* < .001 for all). In addition, Tp-e also significantly correlated with SV (*r* = −0.13394, *P* = .0019) and CO (*r* = 0.4628, *P* < .0001).

**Table 5:**
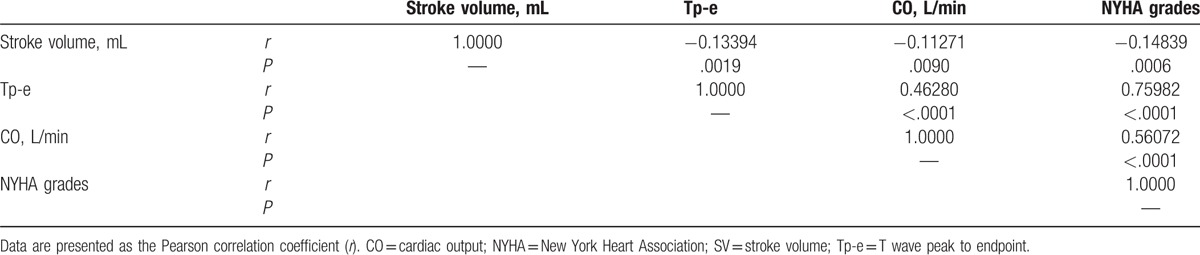
Results of Pearson analysis of correlations between New York Heart Association class and the stroke volume, cardiac output, and T wave peak to endpoint parameter of ischemic cardiomyopathy patients.

## 4 Discussion

In the present study, we examined whether a combination of demographic and clinical characteristics and EKG and UCG parameters correlated with cardiac functional status in elderly Chinese patients with ICM. The multivariate logistic regression analysis of the UCG and EKG variables showed that the disease course, HR, Tp-e, QTd, FS, and CO were significantly associated with the NYHA class (*P* < .05, Table 4), and a Pearson correlation analysis showed that Tp-e, SV, and CO were significantly correlated with the NYHA class (*P* < .05, Table 5). Considering that only Tp-e and CO varied significantly between all the NYHA classes, exhibiting distinct increasing trends with increasing NYHA class (Table 2) regardless of T2DM status (Tables S1 and S2), we propose that an index based on Tp-e and CO might be useful for corroborating the results of the NYHA functional assessment of ICM patients.

Although the NYHA classification is widely used for assessing the functional status of patients with cardiac disease, differences in physicians’ interpretations of the NYHA criteria contribute to substantial variation in the assignment of the functional class, with one previous study reporting that physicians’ evaluations of the NYHA criteria were in agreement only 53% of the time.^[6]^ To overcome this high level of variation, previous studies have examined the benefits of using more objective clinical evaluations of patients with cardiac disorders through comparisons of the NYHA class with assessments of cardiac performance, including the 6-minute walk, anerobic threshold, and peak VO_2_.^[7,26–29]^ However, these methods also display a substantial degree of variability, with Rostagno et al^[7]^ reporting a high level of discordance between the results of various assessments for the same patient. Although studies have reported satisfactory reproducibility for the 6-minute walk test, the results were sensitive to patient perceptions.^[8,30]^

The value of EKG parameters in assessing patients with ischemic heart disease has been demonstrated since the early 1970s,^[31–34]^ with more recent studies focusing on the role of ventricular repolarization and TDR in the manifestation of the subsequent pathology^[19,35–39]^ in patients with and without diabetes.^[34,40]^ We know of no previous comprehensive comparison of the NYHA assessment and TDR in ICM patients. However, there have been several studies in which HR variability changes were reported to be significantly different between NYHA class patients.^[41,42]^ We observed differences in the HR and SV of ICM patients between the NYHA classes, which is in agreement with published reports.^[43,44]^ Previous studies have also shown that UCG is useful for objectively evaluating left ventricular systolic and diastolic function^[45]^ as well as assessing overall cardiac performance.^[14]^ Our observation that Tp-e correlated with CO represents a link between cardiac electrophysiology and cardiac function, and warrants future studies of the correlative relationships between EKG parameters and the results of assessments for cardiac performance and exercise capacity.

The interpretation of our results is subject to certain limitations. Owing to the relatively small number of patients in each NYHA group and T2DM subgroup, a more powerful statistical analysis, such as the estimation of odds ratios using Cox regression, was not performed. In addition, Tp-e is not a direct measure of cardiac performance or functional status. However, our observation that Tp-e was associated with the NYHA class of ICM patients is consistent with the findings of previous studies, which reported that perturbations of the QT interval were associated with variation in the extent of deterioration of cardiac function.^[34,39,40]^ In addition, UCG is not the most accurate and reliable method for determining CO. For inpatients at our hospital, we consider serum brain natriuretic peptide levels and ICM diagnostics combined with EKG measurements to determine CO. However, more accurate methods of determining CO are not available in some outpatient clinics. Future research is warranted to investigate whether CO determined using other methods is also associated with NYHA class. Moreover, alternatives to calculate the repolarization time and dispersion rate of cardiac myocytes could be the element free Galerkin, meshless local Petrov Galerkin, or radial basis function-based methods^[46,47]^.

## 5 Conclusions

Our results suggest that an index consisting of Tp-e and CO might be useful as an objective means of corroborating the results of NYHA assessments of ICM patients. Future studies of the relationship between EKG and UCG parameters and NYHA-based functional assessment will be required to confirm our findings.

## Supplementary Material

Supplemental Digital Content
