# Supplementary material for: Echocardiography and Electrocardiography Variables Correlate With the New York Heart Association classification: An Observational Study of Ischemic Cardiomyopathy Patients
Source: Medicine (Baltimore). 2017 Jun 30;96(26):e7071. doi: 10.1097/MD.0000000000007071 (PMC5500022; doi:10.1097/MD.0000000000007071)
Supplement: Supplemental Digital Content [file medi-96-e7071-s001.doc]

Figure S1. Data plots for Pearson correlation analysis

**
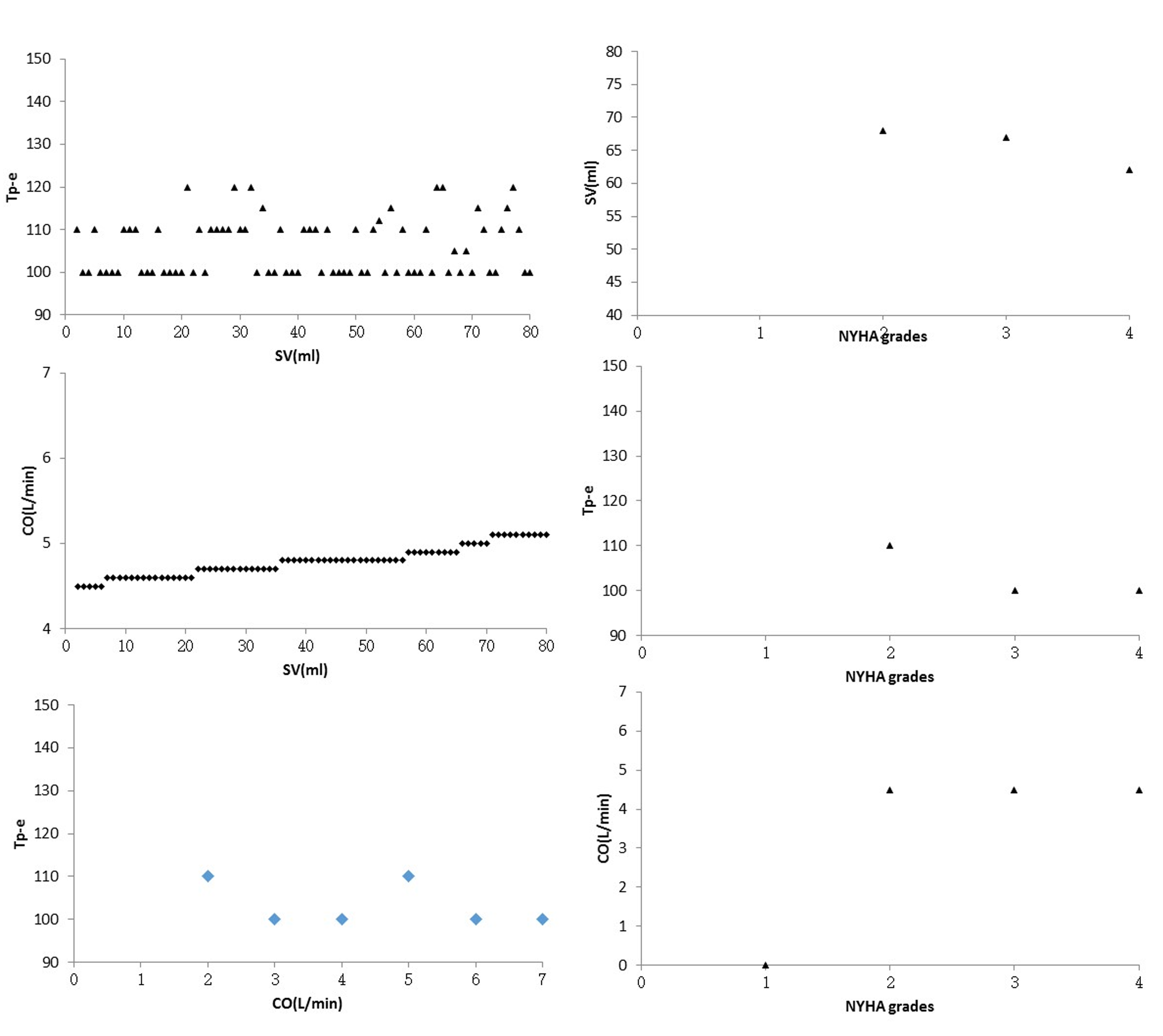
**

Table S1. Comparison of electrocardiography variables between New York Heart Association classes based on type 2 diabetes status

|  | Tp-e (µs) | QTc (µs) | QTd (µs) | Tp-e/QTc |
| --- | --- | --- | --- | --- |
| T2DM (n = 196) | | | | |
| Class I | 100 ± 10^a^ | 400 ± 10^a^ | 30 ± 5^a^ | 26 ± 3^a^ |
| Class II | 114 ± 15^b^ | 400 ± 25^a^ | 39 ± 10^b^ | 28 ± 3^b^ |
| Class III | 120 ± 10^c^ | 410 ± 15^b^ | 40 ± 5^b^ | 29 ± 2^b^ |
| Class IV | 130 ± 10^d^ | 410 ± 10^b^ | 45 ± 10^c^ | 32 ± 2^c^ |
| *P*-value* | < 0.0001 | < 0.0001 | < 0.0001 | < 0.0001 |
| Non-T2DM (n = 340) | | | | |
| Class I | 110 ± 10^a^ | 400± 25^a^ | 30 ± 5^a^ | 27 ± 4^a^ |
| Class II | 114 ± 10^b^ | 400 ± 15^a^ | 37 ± 10^a^ | 28 ± 3^b^ |
| Class III | 120 ± 10^c^ | 410 ± 15^b^ | 40 ± 10^b^ | 30 ± 2^c^ |
| Class IV | 130 ± 10^d^ | 410 ± 20^b^ | 46 ± 10^c^ | 32 ± 2^d^ |
| *P*-value* | < 0.0001 | < 0.0001 | < 0.0001 | < 0.0001 |

Data are presented as the mean ± standard error.

Tp-e, T wave peak to endpoint; QTc, corrected QT; QTd, QT dispersion

^a, b, c, d^Values for each variable that have the same letter are not significantly different (*P* > 0.05), whereas different letters indicate a significant difference in the value of the variable between two NYHA classes (*P* < 0.05).

*Comparison of the variable across all NYHA classes.

Table S2. Comparison of echocardiography variables between New York Heart Association classes based on type 2 diabetes status

|  | CO  (L/min) | SV  (mL) | FS  (%) | LVEF  (%) | LVIDd  (mm) | E/A<1  [n (%)] | E/A>1  [n (%)] |
| --- | --- | --- | --- | --- | --- | --- | --- |
| T2DM | | | | | | | |
| Class I | 5.00 ± 0.50^a^ | 57.00 ± 14.00^a^ | 35.00 ± 10.00^a^ | 62.00 ± 7.00^a^ | 47.00 ± 6.00^a^ | 36 (24.5) | 25 (100) |
| Class II | 5.20 ± 0.70^b^ | 64.00 ± 12.00^b^ | 31.00 ± 7.00^b^ | 60.00 ± 5.00^a^ | 47.50 ± 11.00^a^ | 12 (8.2) | 0 (0.00) |
| Class III | 5.65 ± 0.60^c^ | 63.00 ± 8.50^b^ | 27.00 ± 5.00^b^ | 57.00 ± 6.50^b^ | 55.00 ± 9.00^b^ | 45 (30.6) | 0 (0.00) |
| Class IV | 5.80 ± 0.80^d^ | 56.00 ± 9.00^a^ | 27.00 ± 10.00^b^ | 49.00 ± 9.00^b^ | 57.00 ± 8.00^b^ | 54 (36.7) | 0 (0.00) |
| *P*-value* | < 0.0001 | < 0.0001 | < 0.0001 | < 0.0001 | < 0.0001 | < 0.0001 | |
| Non-T2DM | | | | | | | |
| Class I | 5.10 ± 0.40^a^ | 62.00 ± 10.00^a^ | 34.00 ± 8.00^a^ | 64.00 ± 9.00^a^ | 47.00 ± 4.00^a^ | 46 (17.6) | 33 (100) |
| Class II | 5.20 ± 0.50^b^ | 61.00 ± 10.00^a^ | 33.00 ± 6.00^a^ | 62.00 ± 5.00^a^ | 50.00 ± 4.00^b^ | 103 (39.5) | 0 (0.00) |
| Class III | 5.60 ± 0.50^c^ | 63.00 ± 7.00^a^ | 28.00 ± 6.00^b^ | 57.00 ± 5.00^b^ | 54.50 ± 11.00^c^ | 64 (24.5) | 0 (0.00) |
| Class IV | 5.70 ± 0.50^d^ | 55.00 ± 11.50^b^ | 23.00 ± 10.50^c^ | 47.50 ± 9.00^c^ | 56.00 ± 7.50^c^ | 48 (18.4) | 0 (0.00) |
| *P*-value* | < 0.0001 | < 0.0001 | < 0.0001 | < 0.0001 | < 0.0001 | < 0.0001 | |

Data are presented as the mean ± standard error.

SV, stroke volume; LVEF, left ventricular ejection fraction; FS, left ventricular fractional shortening; LVIDd, left ventricular internal diameter at end-diastole; E/A = ratio of the peak mitral blood flow velocity during early diastole to that during atrial contraction; CO, cardiac output

^a, b, c, d^Values for each variable that have the same letter are not significantly different (*P* > 0.05), whereas different letters indicate a
